# Different Scales, Different Rules: How Do Temperature and Radiation Across Macro‐ and Microclimate Shape Forest Insect Traits?

**DOI:** 10.1002/ece3.74027

**Published:** 2026-08-12

**Authors:** Ruth Pickert, Lea Heidrich, Soumen Mallick, Jonas Hagge, Lukas Lehnert, Oliver Mitesser, Soyeon Bae, Inken Dörfler, Martin M. Gossner, Peter Schall, Wolfgang W. Weisser, Jörg Müller

**Affiliations:** ^1^ Chair of Conservation Biology and Forest Ecology, Ecological Station Fabrikschleichach, Faculty of Biology, Biocenter, Theodor‐Boveri Institute for Biosciences Julius‐Maximilians‐Universität Würzburg Würzburg Germany; ^2^ Environmental Informatics, Department of Geography University of Marburg Marburg Germany; ^3^ Northwest German Forest Research Institute Forest Nature Conservation Hann. Münden Germany; ^4^ University of Göttingen Forest Nature Conservation Göttingen Germany; ^5^ Department of Geography Ludwig‐Maximilians‐Universität Munich Germany; ^6^ Institute of Farm Economics Johann Heinrich von Thünen Institute Braunschweig Germany; ^7^ Institute of Biology and Environmental Science, Vegetation Science & Nature Conservation University of Oldenburg Oldenburg Germany; ^8^ Forest Entomology, Swiss Federal Research Institute WSL Birmensdorf Switzerland; ^9^ Institute of Terrestrial Ecosystems, Department of Environmental Systems Science ETH Zürich Zürich Switzerland; ^10^ Silviculture and Forest Ecology of the Temperate Zones. Georg‐August‐Universität Göttingen Göttingen Germany; ^11^ Terrestrial Ecology Research Group, Department for Life Science Systems, School of Life Sciences Technical University of Munich Freising Germany; ^12^ Bavarian Forest National Park Grafenau Germany

**Keywords:** body size, colour lightness, heat conservation, solar radiation, temperature, thermal melanism

## Abstract

Ecophysiological rules such as the *Heat Conservation* and *Thermal Melanism Hypotheses,* the *Heat Transfer Theory* or *Bogert's Rule* predict different relationships between insect body size, colour lightness and thermal environments. Forests provide scale‐dependent thermal conditions – strong macroclimatic gradients across regions and microclimatic buffering depending on canopy density, with dense canopies producing cooler, more stable conditions and open canopies allowing higher light and heat input. Consequently, trait–environment relationships may vary accordingly. Here we test across both macro‐ and microclimatic scales whether temperature and solar radiation shape insect communities across different taxa with distinct activity periods. Location: Five forest regions across Germany. Time period: 2007–2018, non‐continuous sampling. Major taxa studied: Predominantly diurnal Coleoptera and Heteroptera and nocturnal Lepidoptera. We used linear mixed effect models to test the response of mean community values to macro‐ and micro‐climatic variables. We found smaller Coleoptera and smaller and lighter‐coloured Lepidoptera with increasing seasonal mean temperature, in line with the *Heat Conservation* and *Thermal Melanism Hypotheses*. However, increasing solar radiation became increasingly important at microclimatic scales, with consistently smaller and darker‐coloured Coleoptera and darker‐coloured Lepidoptera in forests with open canopies, in line with the *Heat Transfer Theory* and *Bogert's Rule*. Heteroptera showed only a response in body size at the macroclimatic scale. Our findings suggest that cross‐taxon community responses to macroclimate follow different mechanisms than those to microclimate and might be determined by the life history traits of the selected taxa. Further studies on arthropods with different activity periods in forests along microclimate gradients will help to further disentangle these mechanisms. Changes in macroclimate caused by climate change, along with changes in canopy cover at the microclimatic level, make understanding the mechanism how this leads to scale‐dependent environmental trait filtering and consequently affects insect communities increasingly important.

## Graphical abstract

We show that temperature and solar radiation shape insect community traits in scale‐dependent ways across German forests. Whereas macroclimatic temperature predicts consistent shifts in body size and colouration, microclimatic variation driven by canopy structure can override these broad patterns, producing contrasting trait filtering among taxa.

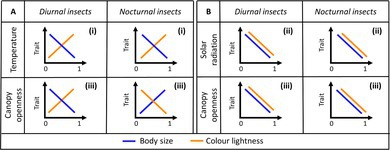

## 1 Introduction

Understanding how organisms respond to climatic variation is a central question in ecology, especially under rapid climate change. Early ecophysiological rules such as Bergmann (1847) or Bogert (1949) still stimulate research because they describe how functional traits such as body size or colouration vary with climatic parameters over large spatial scales (Cadotte et al. 2011). Yet, while broad macroclimate gradients such as ambient temperature shape the general distribution of insects, their local occurrence and abundance can be substantially modified by microclimate (Pincebourde and Woods 2020). For example, in forests, microclimate effects of canopy can outweigh current macroclimate changes by altering buffering capacity (De Frenne et al. 2019; Lumbsden‐Pinto et al. 2025). At larger spatial scales, variation in solar energy is largely expressed through ambient temperature and solar radiation gradients. At the microclimatic scale, however, canopy structure can partially decouple radiation and temperature. Increased canopy openness leads to greater amounts of solar radiation at the forest floor, higher daytime temperatures and more rapid nocturnal cooling compared to closed forests (Latif and Blackburn 2010; Zellweger et al. 2020) thereby amplifying diel thermal fluctuations. This has important consequences for ectotherms such as insects given their dependence on external heat sources (Lettenmaier et al. 2022; Pincebourde and Woods 2020; Verdonck et al. 2025) and may additionally influence physiology, development, behaviour and survival (Greiser et al. 2022; Harvey et al. 2020; Lindman et al. 2022). While macroecological patterns in functional traits have been extensively studied, less is known about whether those ecophysiological rules hold at smaller spatial scales (Woods et al. 2015).

**FIGURE 1:**
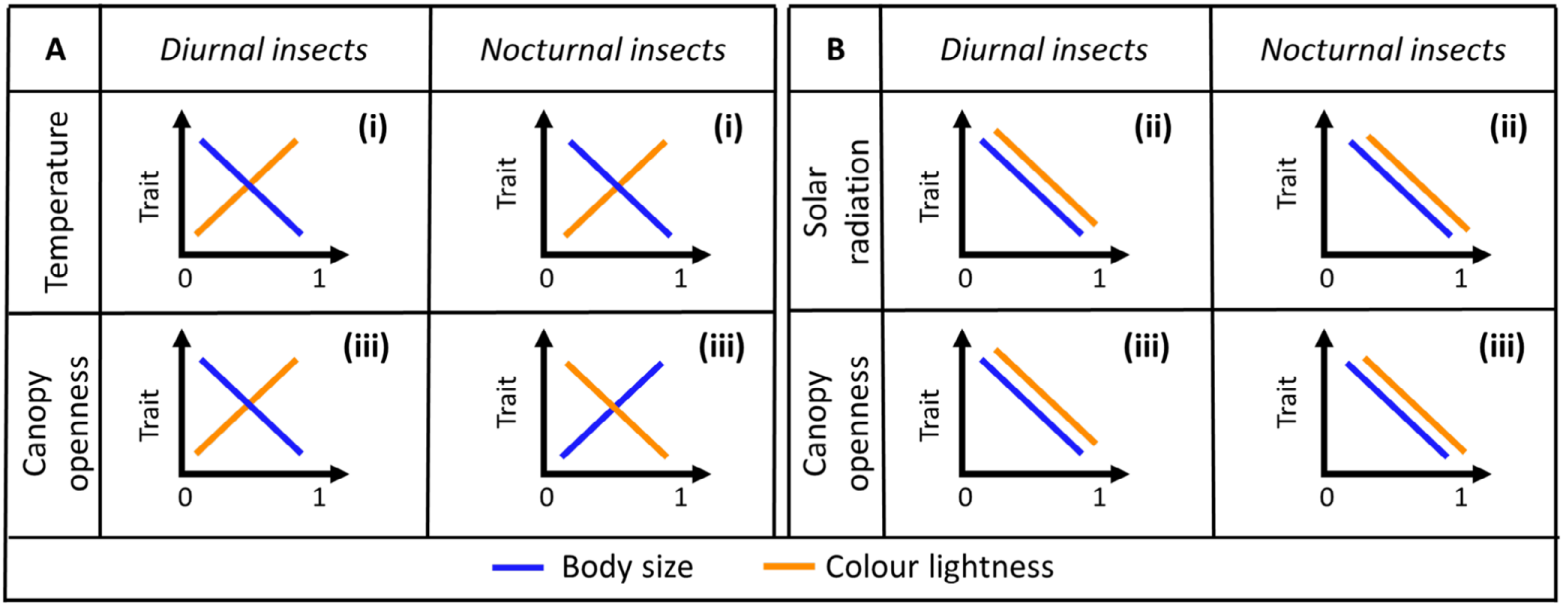
Visual representation of study predictions. According to the *Heat Conservation* and *Thermal Melanism Hypotheses* (A) we predict (i) a decrease in body size (blue) and an increase in colour lightness (orange) with increasing temperature for both diurnal and nocturnal insects, but (A iii) that the local effect of canopy openness leads to opposing patterns for nocturnals. In contrast, we predict a decrease in colour lightness and body size for diurnal and nocturnal insects with both increasing solar radiation (ii) and local canopy openness (B iii), consistent with the *Heat Transfer Theory* and *Bogert's Rule* (B).

Macroecological studies suggest two key morphological traits which are associated with thermoregulation: body size and melanisation. Body size is influenced by temperature through multiple mechanisms. The surface‐area to volume ratio decreases with size, enhancing the ability to conserve heat, as predicted by the *Heat Conservation Hypothesis* (Bishop et al. 2016; Heidrich et al. 2021; Xing et al. 2016; Zamora‐Camacho et al. 2014) (Figure 1A, Figure S1). Consequently, this hypothesis predicts larger body sizes in colder environments. Additionally, this surface‐area to volume ratio also influences the risk of desiccation, with smaller individuals being more vulnerable to desiccate than larger ones (Bujan et al. 2016; Nervo et al. 2021; O'Donnell 2022). Similarly, lower ambient temperatures slow down larval development, allowing individuals to grow longer and reach larger adult sizes, a pattern which is known as the Temperature‐Size‐Rule (Atkinson and Sibly 1997; Ma et al. 2021; Verberk et al. 2021). Yet, the rate of heat gain may play a more crucial role than heat retention (Alcantara et al. 2024). Small body size may be advantageous in colder environments by allowing quicker heat gain and therefore increased activity due to reduced thermal inertia (Blanckenhorn and Demont 2004; Shelomi 2012). Together, these mechanisms imply that both larger and smaller bodies can be favoured in colder environments, depending on whether heat conservation or rapid heat gain dominates. However, in this study, we primarily adopt the *Heat Conservation Hypothesis* and therefore hypothesise that lower temperatures are associated with larger body sizes (Figure 1A).

In addition to temperature‐based mechanisms, solar radiation represents a distinct driver of body size variation through the *Heat Transfer Theory*, based on the theoretical framework of Stevenson (1985). In contrast to the mechanisms outlined above, which primarily consider ambient temperature effects on development and heat balance, this theory explicitly focuses on biophysical heat exchange processes. Larger ectotherms have a greater surface area exposed to sunlight and thus absorb more solar radiation, but also dissipate heat more slowly through convection, making them more prone to overheating and UV damage (Clusella‐Trullas et al. 2007; Porter and Gates 1969; Stevenson 1985). Consequently, smaller body sizes may be favoured in environments with high solar radiation, where rapid heat dissipation is advantageous (Figure 1B, Figure S2).

The second key trait, colouration, is also predicted to respond to temperature and solar radiation at the macroecological scale. The *Thermal Melanism Hypothesis* predicts darker pigmentation in colder climates, enhancing heat absorption and thus enabling ectotherms to reach their optimal temperature for activity more quickly (Bishop et al. 2016; Clusella‐Trullas et al. 2007; Heidrich et al. 2018; Zeuss et al. 2014) (Figure 1A, Figure S1). Although fitness benefits may vary between taxa (True 2003), there is considerable support for this hypothesis in different insect orders (e.g., in Bishop et al. 2016; Pinkert et al. 2017; Stelbrink et al. 2019), even in nocturnal insects, suggesting that there might be a pleiotropic link between ambient temperature and colouration apart from thermoregulatory benefits (Heidrich et al. 2018; Xing et al. 2018). Conversely, *Bogert's Rule*, also referred to as the UV‐Protection Hypothesis (Badejo et al. 2020; Xing et al. 2018) or Photo‐Protection Hypothesis (Law et al. 2020), proposes darker pigmentation in areas of high solar radiation, due to the photoprotective and desiccation‐resistant properties of melanin (Battisti et al. 2013; Bishop et al. 2016; Heidrich et al. 2018; Roulin 2014; Xing et al. 2023) (Figure 1B, Figure S2). These mechanisms may act in synergy or conflict, depending on whether temperature (*Thermal Melanism Hypothesis*) or solar radiation (*Bogert's Rule*) is the dominant selective force.

At the microclimatic scale in forest habitats, canopy openness alters both the thermal and radiation environment insects experience (Woods et al. 2015), thereby possibly acting as an additional environmental filter for thermal traits (Pincebourde and Woods 2020). The canopy‐induced differences in thermal dynamics and UV radiation interact with insects' activity periods may create contrasting trait optima for body size and colouration. While diurnal insects may experience solar radiation directly during activity, nocturnal insects are only passively exposed to solar radiation during daytime resting periods. This may influence physiological processes such as UV resistance, desiccation tolerance and oxidative stress, but not thermoregulatory mechanisms relevant for activity (Heidrich et al. 2018; Xing et al. 2018). However, research on microclimatic effects on climate‐sensitive traits is still rare, and even fewer studies have examined more than one taxonomic order, that show different activity patterns and thermal strategies (Law et al. 2020).

In this study, we tested four ecological hypotheses across two spatial scales—macroclimatic and microclimatic contexts. Specifically, we examined the temperature‐related mechanisms proposed by the *Heat Conservation* and *Thermal Melanism Hypotheses*, and the radiation‐based mechanisms described by the *Heat Transfer Theory* and *Bogert's Rule* (Figure 1). Specifically, we addressed two central questions:

1. How do large‐scale variations in ambient temperature and solar radiation, both acting as coarse‐grain environmental filters, shape body size and colouration in forest insect communities across broad geographic gradients?
2. How does variation in local canopy structure affect trait composition, filtering at the microclimatic scale, and to what extent are these effects shaped by thermal and radiation conditions experienced during different activity periods?

To answer these questions, we analysed how ambient temperature and solar radiation, both measured at macro‐ and microclimatic scales, shape the functional trait composition of insect communities in temperate forests. Specifically, we assessed body size and colouration across three larger insect groups: on predominantly diurnal Coleoptera and Heteroptera as well as nocturnal Lepidoptera. Our approach combined high‐resolution canopy density information derived from Airborne LiDAR with annual mean temperature and solar radiation, allowing us to disentangle the relative contributions of microclimate and macroclimate, respectively. By including both diurnal and nocturnal taxa, we were able to account for differences in exposure to solar energy and thermal regimes associated with diel activity.

Based on our four focal hypotheses (Figure 1), we predict that insect communities, independently of their activity period, will show smaller body size and lighter colouration with increasing macroclimatic temperature (prediction (i)), while high solar radiation may promote smaller and darker species (prediction (ii)).

On the plot level (prediction (iii)), greater solar exposure in open‐canopy sites may favour light colouration and select for smaller body size of diurnal individuals to minimise overheating. In contrast, nocturnal insects, active during the cooler night, may benefit from larger body sizes that help retain heat under more rapidly cooling conditions. At the same time, increased UV radiation can favour darker pigmentation due to the protective function of melanin. Because radiation affects insects during both active and resting phases, these responses are largely independent of diel activity pattern, with smaller and darker‐coloured individuals favoured in more open‐canopy conditions regardless of activity period. For diurnal species, this creates a trade‐off between avoiding overheating and ensuring adequate UV protection, whereas nocturnal species primarily experience the UV‐driven component.

## 2 Methods

### 2.1 Study Area and Insect Sampling

**FIGURE 2:**
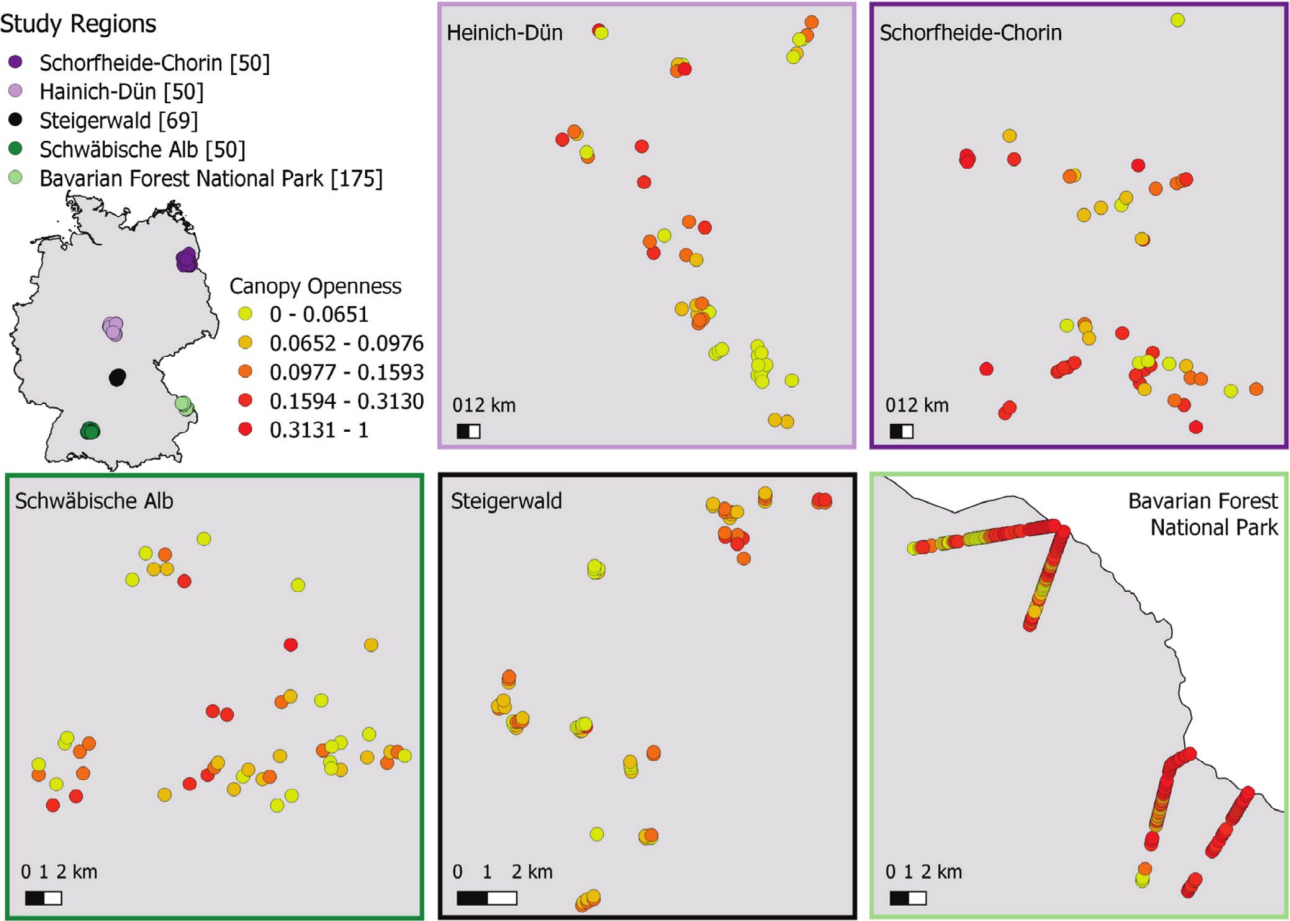
Study regions across Germany included in this analysis. The inset map (upper left) shows the geographic location of the five study regions within Germany. The main panels display the regions of the Biodiversity Exploratories (Schorfheide‐Chorin, Hainich‐Dün, Schwäbische Alb), the Steigerwald Project (Steigerwald) and the BIOKLIM Project (Bavarian Forest National Park). Only plots equipped with at least one trap system and for which environmental parameters were available are displayed. Within each region, plots are colour‐coded according to canopy openness, grouped into quintiles, ranging from 0 (completely closed canopy = yellow) to 1 (completely open canopy = red), with a continuous gradient reflecting increasing openness.

Our study is based on data compiled in Bae et al. (2021) (dataset reference: Bae et al. 2019a) and Heidrich et al. 2020, which were acquired in five forest regions in Germany (Figure 2) that were included in three scientific projects: the Biodiversity Exploratories Project (Fischer et al. 2010) covering three regions (from north to south): UNESCO Biosphere Reserve Schorfheide‐Chorin (N 52° 86′–53° 19′; E 13° 63′–14° 00′), UNESCO Biosphere Reserve Hainich‐Dün (N 51° 05′–51° 37′; E 10° 21′–10° 53′), UNESCO Biosphere Reserve Schwäbische Alb (N 48° 36′–48° 50′; E 9° 20′–9° 50′) with 50 plots each; the Steigerwald Project (Doerfler et al. 2017) in northern Bavaria (N 49° 80′–49° 94′; E 10° 45′–10° 62′), with 69 plots and the BIOKLIM Project (Bässler et al. 2009) located in the Bavarian Forest National Park (N 48° 91′–49° 20′; E 13° 19′–13° 45′), where 175 plots were equipped with at least one trap system and had complete environmental data available, resulting in a total of 394 plots included in the present study across all regions.

Different types of traps were used to collect the insects between 2007 and 2018 according to standardised protocols. However, sampling was not continuous across all regions: Ground‐dwelling and flying Coleoptera (Gossner et al. 2022) and Heteroptera (Gossner et al. 2023) were collected using pitfall and flight interception traps throughout the periods of insect activity peaks of the study areas. They were recorded in the BIOKLIM Project in 2007, in the Biodiversity Exploratories Project in 2008 and in the Steigerwald project in 2016, respectively. Lepidoptera (Weisser et al. 2021) were collected using light traps on a subset of 277 plots over all study regions in 2018, with each plot sampled twice per plot during the phenological peak. Details on insect sampling can be found in the Supporting Information.

### 2.2 Morphological Trait Values

Colouration was analysed in standardised photographs from literature‐based collections of the full dorsal area of the species using digital image analysis, ensuring consistent conditions across photographs per order, recorded in the wavelength range of visible light (400–700 nm). For Coleoptera, digital photographs were provided by the publisher of ‘Käfer Mitteleuropas’ (Dries 2016). The photograph library ‘Corisa’ (Strauss and Simon 2020) was used for Heteroptera, and the monograph ‘The Macrolepidoptera of Germany’ (Segerer et al. 2011) for Lepidoptera. Due to the inherent differences in the settings of the photographs between the trait sources, direct comparisons are not feasible. Importantly, comparisons within each order, however, are possible due to the standardised recording method within one source. After the removal of the white background, the arithmetic mean of red, green and blue colour was calculated per pixel, with values ranging from 0 (completely black) to 255 (completely white). See Heidrich et al. 2018 and Zeuss et al. 2014 for detailed information on the digital image analysis. Body size was measured as the full body length of the animal, from the head to the last abdominal segment. Body size data were available for all 1622 Coleoptera species, while colour lightness was missing for 67. As both traits were analysed separately, these species remained in the dataset. For Heteroptera, both traits were available for all 183 species. For Lepidoptera, one species was excluded because both traits were missing, leaving 454 species in the dataset. The body sizes of Coleoptera and Heteroptera were taken from the literature (Gossner et al. 2015). The body size of Lepidoptera was digitally measured on the specimens depicted in ‘The Macrolepidoptera of Germany’ (Segerer et al. 2011). Note that the body size traits extracted from literature are mean species values, thus integrating intraspecific variability, whereas the measurements extracted from photographs are means of the specimens depicted there, though they are usually reflective of the variability found in nature (see Figures S3 and S4). However, the potential for intraspecific variability in body size and colouration due to phenotypic plasticity could have a significant impact, given that environmental factors such as ambient temperature and solar radiation are known to drive changes in these traits. Rodrigues and Beldade (2020) emphasise that thermal plasticity enables insects to adjust to local climate conditions, with specimens of the same species developing distinct morphologies in response to varying temperatures and radiation levels. Such phenotypic plasticity introduces variability within species, which may impact model accuracy if not accounted for, particularly in estimating how traits are shaped by environmental factors. Nevertheless, in consideration of the potential influence of phenotypic plasticity, in this study we concentrate on mean species values, thereby ensuring consistency across taxa and datasets, which enables us to identify broad‐scale trends of insect communities on different spatial scales. The species included in our dataset are generally widespread, with endemism being extremely low in Central Europe (Essl et al. 2013). The use of species mean values is standard in macroecological studies on trait‐environment relationships (Zeuss et al. 2014). To validate this approach, we compared individual measurements with species mean values for all Lepidoptera in our dataset (454 species, 1947 specimens, body size: *R* ^2^ = 0.96; colour lightness: *R* ^2^ = 0.90, see Figures S3 and S4 with *Biston betularia* (Figure S5) highlighted as species famous for its intraspecific variation), and for an independent dataset of saproxylic Coleoptera (176 species, 378 specimens, body size: *R* ^2^ = 0.96, see Figure S6), demonstrating that species means can reliably represent individual traits and highlighting the robustness of this approach. Detailed information on insect traits including summarising statistics and trait correlation is provided in the Figure S7.

### 2.3 Accounting for Phylogenetic Relatedness

Underlying evolutionary constraints may shape trait development and diversification, particularly among closely related species that have evolved under similar environmental conditions (Poff et al. 2006). We therefore examined also the phylogenetic relationships within the insect groups using phylogenetic trees for Coleoptera (Müller et al. 2019), Heteroptera (Heidrich and Müller 2023b) and Lepidoptera (Heidrich and Müller 2023a). We partitioned the traits into a phylogenetic and specific component using Lynch's method (Lynch 1991) with linear models for each order and trait separately. The phylogenetic component represents the variation attributable to shared evolutionary history, whereas the specific component reflects variation independent of phylogeny, likely shaped by ecological selection (see Acquah‐Lamptey et al. 2020 and Zeuss et al. 2014 and the Supporting Information for detailed information on phylogenetic and specific components of trait values).

### 2.4 Climate and Radiation Data

We collected the following environmental variables for each sampling plot: Data on macroclimatic temperature conditions were collected as part of the WorldClim project (Hijmans et al. 2025) in 1 km resolution grid. WorldClim provides long‐term 30‐year climate normals (1970–2000) that capture stable climatic patterns rather than short‐term fluctuations, making it particularly suitable for assessing broad‐scale ecological and evolutionary patterns. Moreover, its widespread use in ecological research ensures comparability with many ecological studies using the same dataset. To refine estimates, we applied a 1 km buffer around each plot, weighting temperature by the proportional overlap of grid cells. This accounts for spatial variation and avoids single‐cell bias. While macroclimatic conditions shape insect communities year‐round (Müller et al. 2024), we focused on the mean temperature during their main activity and trapping period (May–August), termed ‘seasonal temperature’. In our dataset, seasonal temperature ranged from 10.8°C to 16.2°C. While we acknowledge that seasonal temperatures and phenological timing may vary among years, our analysis focuses on spatial trait–environment relationships across sites. Thus, the results should be interpreted as reflecting general macroecological patterns rather than year‐specific variation in species activity or sampling windows. Although seasonal temperature was moderately correlated with latitude (Pearson's *r* = 0.66, *p* < 0.001), we retained seasonal temperature as the focal predictor because it directly reflects the mechanism of interest. Latitude was not included, as this gradient may additionally capture continental climatic effects beyond temperature alone, but was accounted for by including region as a random effect (see section Statistical analysis). To measure macroclimatic solar radiation at the top of the canopy, sun's position, local topography and cloud frequency, derived from the MODIS products (Platnick et al. 2017) were considered in a 1 km resolution, as these factors are associated with latitude and elevation. Solar radiation ranged from 322.7 to 396.1 Wm^−2^. Details on the calculation of solar radiation are provided in the Figure S8. Canopy openness, as driver of microclimatic conditions, was used as a proxy (Seibold et al. 2016) and was determined from airborne laser scanning data, collected between 2007 and 2018 depending on the region, in a fine‐grained resolution with eight to 40 pulses per 1 × 1 m raster cells, aggregated to the 100 × 100 m plot size. The canopy cover was also determined based on the penetration ratio of the canopy and the understory layer (above 2 m), with 0 and 1 representing completely closed and open canopies, respectively (see Bae et al. 2019b for details on canopy openness). Among the selected environmental factors, a negative correlation (−0.59) was found between canopy openness and seasonal temperature (Figure S9), and between solar radiation and seasonal temperature (0.82) (see also Figure S10, for density histograms of the environmental variables). To address the latter multicollinearity, we derived the residuals of solar radiation after regressing it against seasonal temperature, prioritising temperature as the physiologically more proximal driver for development, survival, voltinism or species turnover (Abarca and Spahn 2021; Bale et al. 2002; Colinet et al. 2015; Gaytán et al. 2022). Hence, we used seasonal temperature and residual solar radiation as macroclimate variables and canopy openness as microclimatic predictor.

### 2.5 Statistical Analysis

All data analyses were conducted in R Version 4.4.2 (R Core Team 2024). First, we calculated the sample coverage for each taxon using the ‘*iNEXT3D’* function from the ‘iNEXT.3D’ package (Chao and Hu 2025). Because sample coverage varied among taxa, we based all subsequent analyses on presence‐absence data rather than abundance data. This approach reduces the influence of unbalanced sampling effort, the potential overrepresentation of a few highly abundant species, and inter‐annual population dynamic signals. Further information and summary statistics are provided in the Tables S1 and S2. We used linear mixed effect models from the package ‘lme4’ (Bates et al. 2021) to model variation in mean body size (henceforth ‘body size’) and mean colouration (henceforth ‘colour lightness’) of species at the plot level. For each plot and taxonomic order, we calculated the mean trait value (body size or colour lightness) across all species detected in that plot. To account for differences in the number of species per plot and taxonomic order, we weighted all models by the number of species detected in that plot‐order combination, so that plots with more species contributed proportionally more information to the model estimates. As fixed effects, we included the environmental variables seasonal temperature and residual solar radiation as macroclimate predictors, and canopy openness as microclimate predictor (all linearly standardised between zero and one) and allowed their effects to vary among taxonomic orders by including interaction terms between each environmental variable and order. Region and plot (nested within region) were included as random effects to account for the hierarchical sampling design. This modelling strategy resulted in six distinct models: one each for the morphological, phylogenetic and specific components of body size and colour lightness: lmer(Trait ~ Order + Order: Seasonal temperature + Order: Residual solar radiation + Order: Canopy openness + (1|Region/Plot), weights = nspec).

## 3 Results

### 3.1 Macroclimatic Scale: Temperature and Residual Solar Radiation Elicit Contrasting Trait Responses

Across all six models, explained variance ranged from 29% to 35% for the morphological traits (Table S3) and 39%–56% for the phylogenetic component (Table S4), whereas the specific component showed considerably lower explanatory power, with *R* ^2^ values between 2% and 5% (Table S5).

**FIGURE 3:**
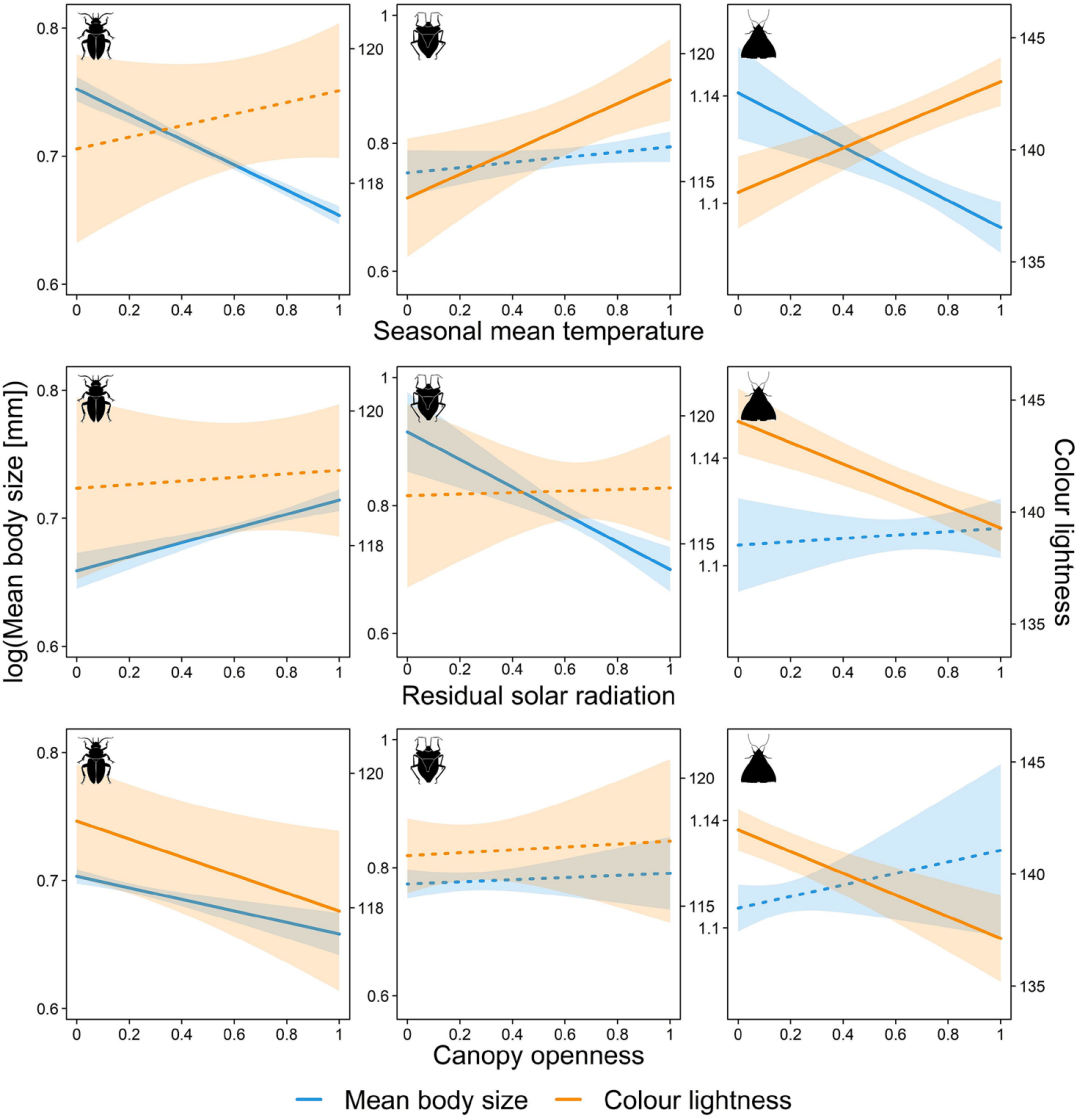
Partial effects derived from linear mixed‐effects models showing the relationships between environmental variables and insect functional traits across taxonomic groups. Shown are the effects of seasonal mean temperature (top row), solar radiation (middle row) and canopy openness (bottom row) on log‐transformed mean body size (blue) and colour lightness (orange) of Coleoptera (left column), Heteroptera (middle column), and Lepidoptera (right column). Solid lines indicate statistically significant relationships, dashed lines denote non‐significant effects. All effects are shown as partial model predictions while holding other predictors constant.

**FIGURE 4:**
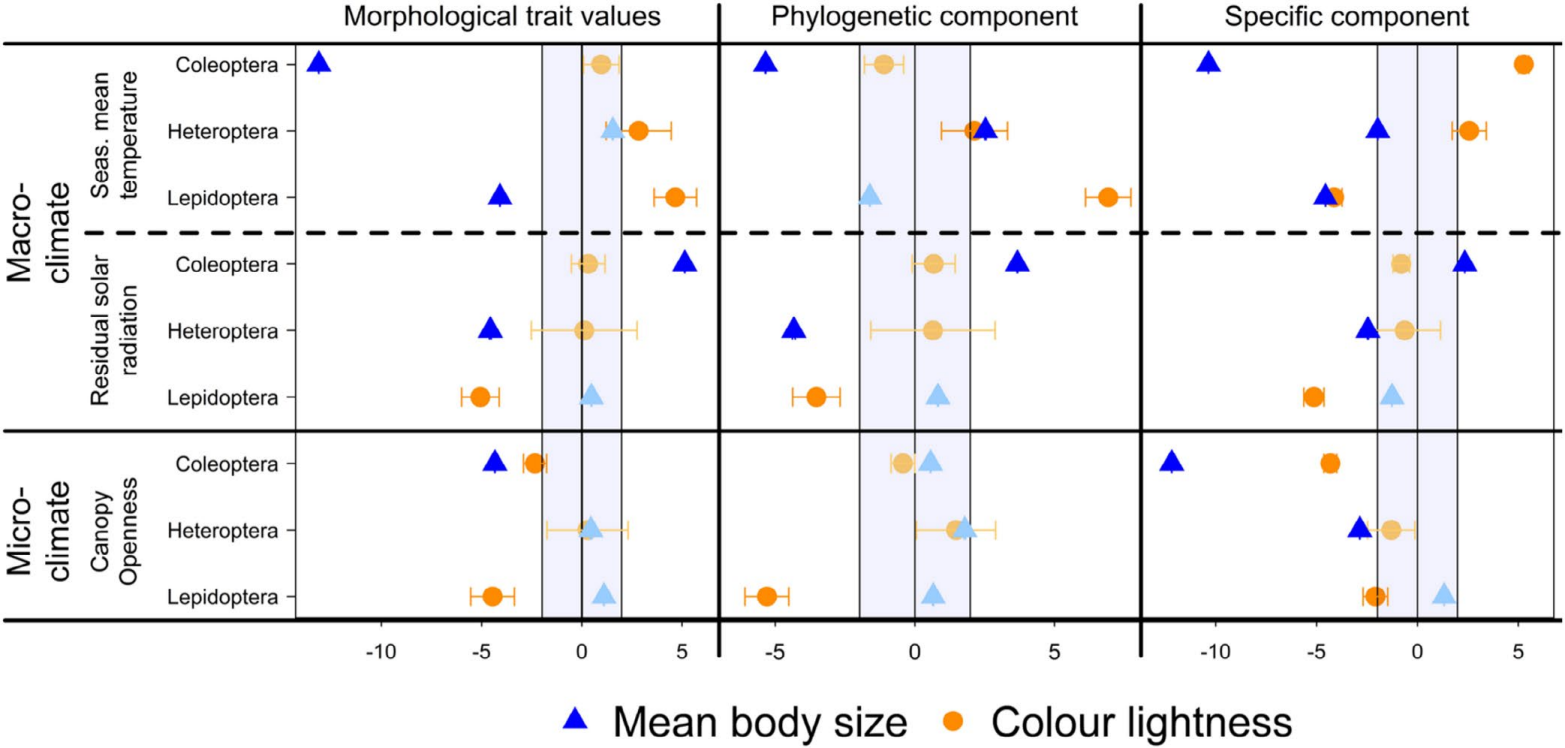
Responses of the phylogenetic component (middle), and the specific component (right) of insect communities to predictor variables representing climatic variables at different scales; the responses of morphological trait values (left) are displayed for direct comparison between phylogeny and morphology. Blue triangles represent mean body size and orange circles represent colour lightness. Significant effects (*p*‐values < 0.05) are displayed in a higher colour‐intensity while light‐coloured effects inside the shaded areas indicate non‐significant values (*t*‐values: ±2.0) Horizontal error bars represent the standard error of the *t*‐values.

The macroclimatic increase in seasonal temperature was associated with divergent trait responses among insect groups. In Coleoptera and Lepidoptera, insects became smaller as temperatures rose (Figure 3, top row; responses of raw trait values can be found in Figure S11). While no significant colour changes were observed in Coleoptera, heteropteran and lepidopteran communities became lighter‐coloured under warmer conditions (Figure 3). These relationships were further clarified by examining the trait components. The pattern of body size change with temperature was consistent in the trait component of Coleoptera in the phylogenetic component (Figure 4). Contrary, Heteroptera became larger in the phylogenetic component (Figure 4). Additionally, all insect groups became smaller in the specific component (Figure 4). Regarding colour patterns with increasing temperature, while Coleoptera became lighter‐coloured only in the specific component, Heteroptera became lighter‐coloured in both the phylogenetic and specific components (Figure 4). Lepidoptera showed contrasting patterns, with lighter colouration in the phylogenetic but darker colouration in the specific component (Figure 4).

Residual solar radiation also caused contrasting trait responses across taxa. High residual solar radiation was associated with larger body sizes in Coleoptera, but with reduced body sizes in Heteroptera. Lepidopteran communities showed no changes in mean body size (Figure 3, second row). In terms of colour lightness, Coleoptera and Heteroptera both did not respond morphologically, but Lepidoptera became darker‐coloured with increasing residual solar radiation (Figure 3, second row). Trait component analysis revealed the exact same patterns: Coleoptera became larger, and Heteroptera smaller under high residual solar radiation for both the phylogenetic and specific components (Figure 4). Additionally, Lepidoptera became darker coloured for both the phylogenetic and specific components (Figure 4).

### 3.2 Microclimatic Scale: Canopy Openness Drives Trait Changes in Coleoptera and Lepidoptera

Canopy openness was associated with divergent trait responses among insect groups. The morphological traits were significantly affected in Coleoptera and Lepidoptera but had no effects on Heteroptera. Diurnal Coleoptera were largest and lightest‐coloured under closed canopy conditions, becoming progressively smaller and darker‐coloured as canopy openness increased (Figure 3, bottom row). Likewise, nocturnal Lepidoptera became darker‐coloured with increasing canopy openness (Figure 3, bottom row). Trait component analysis revealed more nuanced patterns. The specific component of body size in diurnal Coleoptera also became smaller and darker‐coloured under increased canopy openness (Figure 4). For diurnal Heteroptera, the specific component of body size became smaller, despite no morphological response. For nocturnal Lepidoptera, both the phylogenetic and specific components of colour lightness showed the same darkening trend with increasing canopy openness, mirroring the morphological patterns (Figure 4).

## 4 Discussion

Our analysis demonstrates that climate‐related energy inputs, specifically the effects of temperature and solar radiation, differ markedly across spatial scales, insect orders and diel activity periods. Although morphological trait responses varied among insect orders, the phylogenetic and specific components often showed the same directional changes as the morphological patterns under macroclimatic conditions, indicating that it acts as a filter through both historical lineage turnover and contemporary species‐level shifts. Our results show that thermal and radiative pressures shape insect trait distribution in ways that are deeply contingent on spatial scale and ecological context.

### 4.1 Study Limitations

Several limitations should be acknowledged: first, solar radiation was modelled above‐canopy rather than at ground‐level where insects were sampled, potentially overestimating irradiance and yielding conservative effect sizes. Second, the smaller Heteroptera dataset reduced statistical power for this group, though strong and repeated patterns in Coleoptera and Lepidoptera increase confidence in our broader conclusions. Third, we focused on adult traits, although different developmental stages experience distinct thermal (micro‐) habitats and may follow different climate‐trait relationships (Bale and Hayward 2010; Kemppinen et al. 2024; Kingsolver et al. 2011; Radchuk et al. 2013). However, adult traits are central to ecological interactions such as dispersal or reproduction. Fourth, the photographs captured only the visible spectrum (400–700 nm), excluding the near‐infrared (NIR, 700–2500 nm) wavelengths that make up over half of solar radiation (Stuart‐Fox et al. 2017). Although invisible to animals (Kang et al. 2021; Stuart‐Fox et al. 2017), NIR can influence insect thermoregulation (Munro et al. 2019), meaning colour analyses based solely on visible light face methodological limitations. Munro et al. (2019) showed that butterflies from sunnier climates exhibit higher NIR reflectance, supporting climate‐driven variation in insect melanism. Consequently, colour patterns detected in visible‐light studies (e.g., Heidrich et al. 2018, 2021; Pinkert et al. 2017; Stelbrink et al. 2019; Zeuss et al. 2014) and also in the present study should be interpreted with caution, and integrating NIR could provide a more complete understanding of thermal adaptation.

### 4.2 Macroclimatic Effects: Temperature as a Context‐Dependent Driver

Contrary to our expectations regarding prediction (i) of larger bodies under colder climates, the macroclimatic signal of seasonal temperature on body size was not uniform across all insect groups. Both holometabolous orders, Coleoptera and Lepidoptera, were larger in colder environments, a pattern supporting both the *Heat Conservation Hypothesis* (Bishop et al. 2016; Heidrich et al. 2021; Xing et al. 2016; Zamora‐Camacho et al. 2014) and the *Temperature Size Rule* (Atkinson and Sibly 1997; Ma et al. 2021; Verberk et al. 2021), although Coleoptera have been reported to increase in body size with rising temperatures (Maher and Shelomi 2022). These two processes, thermoregulatory advantage and developmental timing, likely act synergistically. The body size of Heteroptera, however, showed no significant body size response to increased seasonal temperature, defying both our expectations regarding prediction (i) and previous evidence, such as historical declines in body size of predatory bugs associated with global warming (Schuldiner‐Harpaz and Coll 2013) and temperature–size relationships reported for other hemimetabolous taxa (Acquah‐Lamptey et al. 2020). This discrepancy supports the view that the *Heat Conservation Hypothesis* may not universally apply to insects, as the underlying mechanisms and selective pressures appear to vary among taxa (Shelomi 2012; Watt et al. 2010). A possible explanation lies in the developmental differences between holometabolous and hemimetabolous insects. In holometabolous taxa, final body size is determined during the larval stage, in which they grow rapidly with a relatively fixed window for mass accumulation (Manthey et al. 2024). Hemimetabolous insects, by contrast, grow incrementally through successive moults, allowing a more flexible response to environmental conditions. Second, the typical form of Heteroptera differs from the other taxa studied, though individual species or genera may deviate in their appearance: they often have a flattened body shape in contrast to the more cylindrical shape of Coleoptera and Lepidoptera. The generalised form of Heteroptera likely increases heat absorption and dissipation and might increase vulnerability to solar radiation and desiccation.

The body shape might also be responsible for the strikingly different responses of the mean body size of Coleoptera and Heteroptera to solar radiation. While, at a given temperature, coleopteran communities tended to comprise larger species under high residual radiation levels, heteropteran communities, following our prediction (iii) based on the *Heat Transfer Theory*, exhibited the opposite trend. This inconsistent pattern suggests that not all predominantly diurnal insects benefit from smaller body sizes when exposed to increased solar input, the risk of overheating and a potential extent of UV exposure. Compared to Heteroptera, Coleoptera, which have rather thick elytra that provide both mechanical protection and water retention (Goczał and Beutel 2023), might be less susceptible to negative impacts of solar radiation. On the contrary, the additional increase in heat gain may benefit larger Coleoptera in cold environments, which would have a rather low heat influx under low solar radiation.

Notably, residual solar radiation had no effect on the body size of Lepidoptera, contradicting our prediction (iii) for nocturnal insects and the *Heat Transfer Theory*. This suggests that being passively exposed to solar radiation during daytime resting periods may not be strong enough to influence physiological processes such as UV resistance, desiccation tolerance and oxidative stress. Interestingly, however, this predominantly nocturnal group was the only one in which the other functional trait—colouration—responded to not only temperature, but also to residual solar radiation, in line with our prediction (iii) and *Bogert's Rule*, whereas the diurnal groups showed no such response.

In terms of colour, darker pigmentation enhances heat absorption, enabling faster warming under cool conditions (Clusella‐Trullas et al. 2007; Zeuss et al. 2014). The pattern of lighter communities in warmer regions is thus consistent with the *Thermal Melanism Hypothesis*. While melanin remains a key determinant of thermal colouration (Haque et al. 2024), other sources of colour—such as structural elements (Datto‐Liberato et al. 2024; Haque et al. 2024) and non‐melanin pigments (Haque et al. 2024; Lindstedt et al. 2010)—may contribute to physiological differentiation among taxa (Datto‐Liberato et al. 2024; Lindstedt et al. 2010). Consequently, thermoregulatory colour functions are likely context‐dependent and intertwined with other ecological and physiological traits. Recent physiological evidence supports the idea that colour traits based on pigmentation reflect multiple functions linked to temperature beyond thermoregulation. For example, Meng et al. (2025) demonstrated that thermal melanism is closely linked to temperature‐mediated phenoloxidase activity, which is central to both melanin synthesis and immune defence. This suggests that darker pigmentation may be the result of pleiotropic interactions between thermal environments, immune investment and cuticular physiology, indicating that colour variation can encode broader adaptive trade‐offs.

Nocturnal insects represent a particularly interesting case. Although Heidrich et al. (2018) argued that colouration should provide little thermoregulatory benefit at night, they still identified macroecological associations between solar energy and colouration in nocturnal Lepidoptera, indicating indirect or cumulative effects, even if the underlying mechanisms remain unclear. Additional thermoregulatory strategies—such as muscle contraction or reduced wing load for efficient flight—may further modulate these patterns and differ among families according to specific lifestyles (Heidrich et al. 2021; Heinrich 2013). Understanding how such behavioural and structural mechanisms interact with pigment‐based thermoregulation remains essential for elucidating adaptive responses of ectothermic insects to thermal variability (Datto‐Liberato et al. 2024).

### 4.3 Microclimate: Canopy Openness Reveals Fine‐Scale Ecological Filtering

At the microclimatic scale, canopy openness produced consistent trait shifts between diurnal and nocturnal taxa. Open canopies elevate daytime temperatures and radiation exposure while increasing nocturnal cooling (De Frenne et al. 2019; Müller et al. 2015). For diurnal Coleoptera, these conditions likely favour smaller, lighter‐bodied individuals that dissipate heat efficiently, allowing effective thermoregulation and avoidance of overheating under elevated temperature and radiations conditions (Cushman et al. 1993). However, we observed increased melanisation, representing a reversal of the macroclimatic pattern and suggesting that microclimatic conditions can override larger‐scale temperature effects. This pattern is consistent with our microclimatic predictions (iii), where increased local radiation due to open canopies may favour darker colouration, potentially reflecting selection for UV protection, desiccation resistance or immune benefits despite potential thermal costs (Xing et al. 2018; Roulin 2014; Badejo et al. 2020). The co‐occurrence of smaller size and darker colour in open canopies suggests a trade‐off: size reduction may offset increased heat absorption from melanisation, allowing individuals to retain UV resistance without overheating. In contrast, nocturnal Lepidoptera were darker in open canopies, following both predictions (ii) and (iii). This likely reflects thermal retention during cool nights and photoprotection during inactive daytime periods (Heidrich et al. 2018; Xing et al. 2018). Because nocturnals are exposed to radiation passively while roosting, selection may favour darker colouration independent of activity temperature. Additionally, melanin's roles in immunity and both pathogen and desiccation resistance may explain its increase even in the absence of thermal advantages (Badejo et al. 2020; Haque et al. 2024; Roulin 2014; Stelbrink et al. 2019). These multifunctional roles of melanin underscore the need to interpret pigmentation patterns through a broader functional framework.

### 4.4 Conceptual Synthesis: Climate‐Trait Rules Are Scale‐ and Context‐Dependent

Our findings reinforce the idea that trait–environment relationships in insects are not uniform but shaped by interacting gradients of temperature, radiation and habitat structure. Macroclimatic temperature drives broad‐scale trait filtering, consistent with ecophysiological rules such as the *Heat Conservation Hypothesis* and the *Thermal Melanism Hypothesis*. However, these rules break down, or even reverse, at microclimatic scales, where canopy structure creates localised thermal and radiative microhabitats. Disentangling morphological, phylogenetic and species‐specific trait components revealed the timescales over which environmental filtering operates. These findings highlight that trait‐based ecology must account for scale‐dependent processes and phylogenetic constraints. At the microscale, body size responses were primarily expressed in the species‐specific component, indicating that local adaptation, developmental plasticity or fine‐scale turnover among ecotypes shape these patterns. In contrast, colour varied in both phylogenetic and specific components, suggesting that pigmentation reflects both evolutionary lineage effects and local filtering. This asymmetry implies that body size is more locally labile, whereas pigmentation tends to be more constrained and evolutionarily conserved (Acquah‐Lamptey et al. 2020; Zeuss et al. 2014). Finally, the direction of selection on size may vary depending on whether daytime overheating or nocturnal heat loss dominates fitness outcomes (Cushman et al. 1993; Le Lagadec et al. 1998). Such context‐dependence helps explain why trait shifts at micro scales can be both strong and inconsistent across taxa.

### 4.5 Implications for Theory, Conservation and Global Change

Our results highlight that classical trait–climate expectations (e.g., *Bergmann's Rule, Thermal Melanism Hypothesis*) may hold at broad scales but be modified or overridden locally. Trait‐based community models must therefore incorporate spatial scale, diel ecology and habitat structure to avoid misinterpretation. Especially incorporating diel activity is essential as traits that enhance heat dissipation during daytime may be maladaptive at night, and *vice versa*. From a conservation perspective, preserving or enhancing microclimatic heterogeneity, via canopy gaps, mixed‐age stands or topographic mosaics, could buffer insect communities against regional warming. Because local canopy structure can override macroclimatic signals, such heterogeneity may be key to maintaining functional trait diversity. Management interventions (e.g., gap creation) could even be tailored to support particular functional groups but should be evaluated with caution to avoid unintended ecological consequences. From a global change perspective, trait shifts, particularly in size and pigmentation, may serve as early indicators of climate impacts. A coordinated network of forest monitoring sites, incorporating trait surveys, canopy structure and microclimate sensors, could provide sensitive detection of climate‐driven ecological change before community collapse occurs.

## 5 Conclusion

Our cross‐taxon, multi‐scale analysis shows that climatic energy, particularly temperature, shapes insect functional traits in scale‐ and taxon‐specific ways. On a macroclimatic scale, seasonal mean temperature consistently influences both body size and colouration of temperate forest insect communities. However, local climatic effects such as canopy cover can outweigh these macroclimatic patterns, leading to contrasting trait filtering among taxa and sites. To cope with environmental change, insects have evolved diverse strategies anchored in their phylogenetic traits. Consequently, the response to a gradual increase in mean temperature over years will differ from the response to abrupt, disturbance‐driven changes such as gap formation. These differences reflect both evolutionary constraints and the flexibility of ecological filtering. Maintaining or restoring fine‐scale microclimatic heterogeneity—such as by creating mosaic‐like structures across forest topography—can enhance insects' ability to respond rapidly and flexibly to changing conditions. Together, our findings highlight that both evolutionary history and contemporary environmental filtering shape community trait composition. Developing trait‐based models that integrate phylogenetic context, spatial heterogeneity and scale‐dependent mechanisms will be essential for predicting insect responses to climate change. Evidence‐based, trait‐informed forest management that fosters structural and microclimatic diversity offers a promising pathway to buffer insect communities against future climatic stress.

## Author Contributions

**Ruth Pickert:** conceptualization (lead), formal analysis (lead), investigation (equal), visualization (lead), writing – original draft (lead), writing – review and editing (lead). **Lea Heidrich:** conceptualization (equal), data curation (equal), formal analysis (equal), investigation (equal), writing – original draft (equal), writing – review and editing (equal). **Soumen Mallick:** writing – original draft (supporting), writing – review and editing (equal). **Jonas Hagge:** conceptualization (equal), data curation (equal), investigation (equal), writing – original draft (supporting), writing – review and editing (supporting). **Lukas Lehnert:** conceptualization (supporting), data curation (equal), writing – original draft (supporting), writing – review and editing (supporting). **Oliver Mitesser:** formal analysis (supporting), writing – original draft (supporting), writing – review and editing (supporting). **Soyeon Bae:** conceptualization (supporting), data curation (equal), writing – original draft (supporting). **Inken Dörfler:** data curation (equal). **Martin M. Gossner:** data curation (equal), writing – original draft (supporting). **Peter Schall:** data curation (equal), writing – original draft (supporting). **Wolfgang W. Weisser:** data curation (equal). **Jörg Müller:** conceptualization (lead), data curation (equal), formal analysis (equal), investigation (lead), supervision (lead), writing – original draft (equal), writing – review and editing (equal).

## Funding

This research received funding by the Deutsche Forschungsgemeinschaft (DFG Priority Program 1374) ‘Biodiversity‐Exploratories’ (433123530).

## Conflicts of Interest

The authors declare no conflicts of interest.

## Supporting information


**Figure S1:** Schematic overview of the responses of body size and colour lightness to temperature according to the *Heat Conservation* and *Thermal Melanism Hypotheses*.
**Figure S2:** Schematic overview of the responses of body size and colour lightness to solar radiation according to the *Heat Transfer Theory* and *Bogert's Rule*.
**Figure S3:** Relationship between measured body size of individual specimens and mean body size for Lepidoptera. There is a strong correlation between individual body size and species mean (*R* ^2^ = 0.96, *p*‐value < 0.001). The blue line represents the linear regression, red triangles highlight *B. betularia* .
**Figure S4:** Relationship between measured colour lightness of individual specimens and mean colour lightness for Lepidoptera. There is a strong correlation between individual colour.
**Figure S5:** Individuals of *Biston betularia* (18–22) depicted in ‘The Macrolepidoptera of Germany’ (Segerer et al. 2011).
**Figure S6:** Relationship between measured body size of individual specimens and mean body size for saproxylic Coleoptera. There is a strong correlation between individual trait and species mean (*R* ^2^ = 0.96, *p*‐value < 0.001). The blue line represents a linear regression. This dataset was not used in our study but is displayed for demonstration purposes.
**Figure S7:** Morphological trait correlation between mean body size and colour lightness for the 1555 species of predominantly diurnal Coleoptera (left), the 183 species of predominantly diurnal Heteroptera (middle) and the 454 species of nocturnal Lepidoptera (right) detected in the five study regions. The scale of the *y*‐axes is logarithmic.
**Figure S8:** Schematic overview of the calculation of Direct Radiation. MOD06 and MYD06 refer to the corresponding products of the MODIS sensor. The R package ‘terra’ was used to calculate the hourly direct radiation and sun angle values.
**Figure S9:** Correlation plot displaying the relationship between the climatic parameters and the residualised variable solar radiation.
**Figure S10:** Density histogram of scaled seasonal mean temperature (left), scaled direct solar radiation (middle) and scaled canopy openness (right) for the five study regions.
**Figure S11:** Responses of raw trait values (mean body size in blue, colour lightness in orange) to environmental parameters.
**Table S1:** Mean sample coverage, also sample completeness, for each taxon based on iNEXT3D (Chao and Hu 2025).
**Table S2:** Summary statistics (minimum, maximum, mean and standard deviation) of environmental variables and spatial distances among plots within each study region. Variables include inter‐plot distance (km), seasonal mean temperature (°C), direct solar radiation (W m^−2^) and canopy openness.
**Table S3:** Effects of macro‐, and microclimate on morphological trait values logarithmised body size and colour lightness of predominantly diurnal Coleoptera and Heteroptera and nocturnal Lepidoptera. The macroclimatic variable temperature is displayed by seasonal mean temperature, direct radiation is residualised. AIC, Akaike information criterion; SE, standard error.
**Table S4:** Phylogenetic component: Effects of macro‐, and microclimate on predicted trait values body size and colour lightness of predominantly diurnal Coleoptera and Heteroptera and nocturnal Lepidoptera. AIC, Akaike information criterion; SE, standard error.
**Table S5:** Specific component: Effects of macro‐, and microclimate on residual trait values body size and colour lightness of predominantly diurnal Coleoptera and Heteroptera and nocturnal Lepidoptera. AIC, Akaike information criterion; SE, standard error.

## Data Availability

This work is based on data inter alia elaborated by the Arthropods and TreeScape projects of the Biodiversity Exploratories program (DFG Priority Program 1374). All datasets are publicly available in the Biodiversity Exploratories Information System (http://doi.org/10.17616/R32P9Q) and listed in the Reference section. Annotated R code, including the data needed to reproduce the statistical analyses and figures, is available in the Biodiversity Exploratories Information System (DOI: https://doi.org/10.71615/bexis.32121).
