# Supplementary material for: Different Scales, Different Rules: How Do Temperature and Radiation Across Macro‐ and Microclimate Shape Forest Insect Traits?
Source: Ecol Evol. 2026 Aug 12;16(8):e74027. doi: 10.1002/ece3.74027 (PMC13469181; doi:10.1002/ece3.74027)
Supplement: Supplementary file 1 [file ECE3-16-e74027-s001.zip › ece374027-sup-0001-Supinfo01.docx]

## **Supplementary Information**

**Title: Different scales, different rules: How do temperature and radiation across macro- and microclimate shape forest insect traits?**

**Authors: Ruth Pickert, Lea Heidrich, Soumen Mallick, Jonas Hagge, Lukas Lehnert, Oliver Mitesser, Soyeon Bae, Inken Dörfler, Martin M. Gossner, Peter Schall, Wolfgang W. Weisser and Jörg Müller**

**Responses of body size and colouration to solar energy**

In insects, body size and colouration are often associated with climatic variables such as temperature or solar radiation (Heidrich et al., 2021). The *Heat Conservation Hypothesis* predicts a smaller body size (Bishop et al., 2016) (Fig. S1) and the *Thermal Melanism Hypothesis* an increased colour lightness (Clusella-Trullas, van Wyk, & Spotila, 2007) (Fig. S1) at higher ambient temperatures. The *Heat Transfer Theory* predicts a smaller body size (Porter & Gates, 1969) (Fig. S2) and *Bogert’s Rule* a decrease in colour lightness under higher amounts of solar radiation (Battisti, Marini, Pitacco, & Larsson, 2013) (Fig. S2).

**Figure S 1:** Schematic overview of the responses of body size and colour lightness to temperature according to the *Heat Conservation* and *Thermal Melanism Hypotheses*.

**Figure S 2:** Schematic overview of the responses of body size and colour lightness to solar radiation according to the *Heat Transfer Theory* and *Bogert’s Rule*.

**Insect trap details**

In the Biodiversity Exploratories, three pitfall traps with a diameter of 15 cm were randomly placed at three corners of each plot from May to October in 2008 (in some but not all cases, pitfall and flight interception traps had already been installed in April; therefore, incomplete records from April were excluded). Two of them were randomly selected for species determination as “Priority 1” or “Priority2”, which could differ between months. In the Steigerwald Project, only one pitfall trap, with a diameter of 8 cm, was installed near the centre of each plot from March to October 2016. The same pitfall trap type was active in the BIOKLIM Project from April to October 2007. The flight interception traps in all projects were of the same model and placed at a height of ~1.5 m nearby. For all projects, a copper sulphate solution served as the trapping liquid. However, the traps at the Biodiversity Exploratories consisted of two sampling units, one at the top and one at the bottom, whereas those at the other projects had a single sampling unit, at the bottom. For the analyses of the pitfall and flight interception traps in the Biodiversity Exploratories, only “Priority 1” samples were used, to ensure equal sampling sizes between projects. Lepidoptera were sampled in all regions with the same type of trap according to the same protocol using super-actinic UV light traps (12V, 15 Watt), connected to a twilight sensor powered by a 12V, 15AH battery, ensuring the exclusive collection of nocturnal species. The light trap was installed twice per plot during the phenological peak, under favourable weather conditions, between May and August. Lepidoptera were sampled in 2017 in the Steigerwald and BIOKLIM Projects and in 2018 in the Biodiversity Exploratories. In addition, a member of a taxon was not always found on every plot equipped with the respective trap type. Thus, Coleoptera were detected on 394, Heteroptera on 346 and Lepidoptera on 227 plots.

**Morphological trait details and trait correlation**

For Heteroptera, one species that was recorded once was indistinguishable from its sister species (*Micronecta minutissima/griseola*). These species are also listed together in the literature, with a single value for body size. For this reason, colour lightness was determined by calculating the mean value of both species, as they hardly differ in their colouration (*Micronecta mantissa*: 122.97; *Micronecta griseola*: 122.64). For Lepidoptera, one species (*Mythimna scirpi*) occurred only once and was therefore excluded from the dataset, because information on its body size and colour lightness was lacking. Thus, 454 nocturnal Lepidoptera species were analysed in total. For the remaining species, both traits were available.

The body sizes of all 1622 Coleoptera species in the data set could be ascertained, but the colour lightness of 67 species was missing. Nevertheless, these species were retained in the dataset since we examined the responses of body size and colour lightness separately to the environmental parameters. Coleopteran colour lightness spanned 69.5 to 159.4 with a mean value of 118.7 and standard deviation (SD) of 15.3. Body size ranged from 0.6 mm to 37.0 mm (mean 5.3 mm ± 4.4 mm SD). For all 183 Heteroptera species, both body size and colour lightness could be determined. Colour lightness in the latter ranged from 91.6 to 149.8 (mean 115.4 ± 9.6 SD) and body size ranged from 1.5 mm to 9.7 mm (mean 5.9 mm ± 3.01 mm SD). The colour lightness of 454 Lepidoptera species ranged from 83.2 to 209.2 (mean 139.3 ± 26.0 SD) while body size ranged from 5.2 mm to 38.9 mm with a mean of 13.7 mm ± 5.0 mm SD. To verify the robustness of species mean values in macroecological studies, we compared individual trait measurements with species mean values for all Lepidoptera in our dataset (454 species, 1,947 specimens) using the monograph “The Macrolepidoptera of Germany” (Segerer, Hausmann, Behounek, Speidel, & Witt, 2011). One lepidopteran species famous for intraspecific variation is *Biston betularia* (Fig. S5), which is highlighted in our analyses for demonstration purposes. Linear models revealed a R^2^ of 0.96 for body length (Fig. S3) and a R^2^ of 0.90 for colour lightness (Fig. S4) between individual measured traits and species means. To further validate our approach, we conducted an additional analysis using an independent dataset of saproxylic Coleoptera (176 species, 378 specimens). Here, body size demonstrated a R² of 0.96 between individual and species mean values (Fig. S6), reinforcing the reliability of our method.

The morphological trait values body size and colour lightness were not significantly correlated in Coleoptera, whereas in Heteroptera species body size correlated positively with colour lightness. In lepidopteran species, by contrast, body size correlated negatively with colour lightness (Fig. S7).

**Figure S 3:** Relationship between measured body size of individual specimens and mean body size for Lepidoptera. There is a strong correlation between individual body size and species mean (R^2^ = 0.96, p-value < 0.001). The blue line represents the linear regression, red triangles highlight *B. betularia*.

**Figure S 4:** Relationship between measured colour lightness of individual specimens and mean colour lightness for Lepidoptera. There is a strong correlation between individual colour lightness and species mean (R^2^ = 0.90, p-value < 0.001). The blue line represents the linear regression, red triangles highlight *B. betularia*.

**Figure S 5:** Individuals of *Biston betularia* (18-22) depicted in “The Macrolepidoptera of Germany” (Segerer et al., 2011).

**Figure S 6:** Relationship between measured body size of individual specimens and mean body size for saproxylic Coleoptera. There is a strong correlation between individual trait and species mean (R^2^ = 0.96, p-value < 0.001). The blue line represents a linear regression. This dataset was not used in our study but is displayed for demonstration purposes.

**Figure S 7:** Morphological trait correlation between mean body size and colour lightness for the 1555 species of predominantly diurnal Coleoptera (left), the 183 species of predominantly diurnal Heteroptera (middle) and the 454 species of nocturnal Lepidoptera (right) detected in the five study regions. The scale of the y-axes is logarithmic.

**Phylogenetic trait details**

We examined the phylogenetic relationships within the insect groups using phylogenetic trees for Coleoptera, containing all 1622 species (Müller, Heibl, & Heidrich, 2019), Heteroptera, containing 170 of 183 species (Heidrich & Müller, 2023b) and Lepidoptera, containing 453 of 454 species (Heidrich & Müller, 2023a). We computed patristic distances using the ‘adephylo’ package (Jombart et al., 2010) per order, followed by PCoAs to derive phylogenetic eigenvectors representing shared evolutionary history for each trait separately. Using linear models, we partitioned trait variation into a phylogenetic component and a specific component, allowing us to distinguish evolutionary constraints from species-specific adaptations or environmental influences. The specific component, represented by the residuals, captured the variation not explained by phylogeny, indicating species-specific variation due to unique adaptations or environmental influences. While the morphological trait includes both evolutionary history and species-specific adaptations, the phylogenetic component represents the portion of trait variation explained by shared evolutionary history, whereas the specific component reflects variation independent of phylogeny, likely shaped by ecological selection.

**Calculation of solar radiation, environmental variables correlation and density histogram**

Radiation at the canopy level between 6 am and 6 pm was estimated between May and September on an hourly basis. Illumination geometries (sun elevation and aspect, Fig. S8) for the upper and lower left and right pixels were calculated for each time step using the ‘terra’ package (Hijmans et al., 2025) and linearly interpolated to the entire grid. From the DEM, slope and aspect were derived using the ‘GDAL’ library (GDAL/OGR contributors, 2024). Since our sampling plots are partly located in steep terrain, cast shadow occurs in the early morning and late afternoon when the sun elevation is low. To remove the effect of cast shadow on the radiation estimates, cast shadow was calculated for each hour on the 15th of each month. Illumination factors for each pixel were then derived:

$$il=cos\left( sl \right)\cdot sin\left( sh \right)+sin\left( sl \right)\cdot cos\left( sh \right)\cdot cos(asz-asp)$$

where *sl* and *asp* are the slope and aspect angle, *sh* is the sun elevation and *saz* is the sun azimuth angle. The illumination has been calculated for hourly steps between 1st of May until 30th of September and was multiplied with the time-dependent cast shadow map. The illumination was then averaged over the entire time series.

Cloud frequency was derived from the MODIS, MOD06 and MYD06 products using all scenes acquired between May and September over the entire time series (approx. 8000 scenes for each location, Platnick et al., 2017). Direct radiation at the top of the canopy (*ρ_dir,c_*) was then estimated by multiplying the mean illumination (*il*) and mean direct radiation (*ρ_dir_*):

$${\rho_{dir}}_{c}=il\cdot\rho_{dir}$$

A test of the correlation between environmental variables showed a correlation between seasonal temperature and direct radiation (Fig. S9) and a negative correlation between seasonal temperature and canopy openness. To address the multicollinearity, we therefore used the residual solar radiation as a proxy for solar radiation.

Fig. S10 shows a density histogram for all environmental variables, scaled from 0 to 1. There is substantial variation in seasonal temperature within the region Bavarian Forest National Park, in a range of about 60% of overall seasonal mean temperature variation.

**Figure S 8:** Schematic overview of the calculation of Direct Radiation. MOD06 and MYD06 refer to the corresponding products of the MODIS sensor. The R package ‘terra’ was used to calculate the hourly direct radiation and sun angle values.

**Figure S 9:** Correlation plot displaying the relationship between the climatic parameters and the residualised variable solar radiation.

**Figure S 10:** Density histogram of scaled seasonal mean temperature (left), scaled direct solar radiation (middle), and scaled canopy openness (right) for the five study regions.

**Additional information for the statistical analyses**

Mean sample coverage (Tab. S1) for all plots was calculated for each taxon separately based on the ‘iNEXT3D’ package (Chao & Hu, 2025). Values close to one indicate nearly complete coverage, suggesting that almost all species that could be present have been observed. Table S2 shows summarising statistics for the environmental variables. Fig. S11 displays the responses of raw trait values to environmental variables. Tables S3, S4, and S5 show the results of the linear mixed effect models for the morphological trait values (Tab. S3), the phylogenetic component (Tab. S4), and the specific component (Tab. S5).

**Table S 1:** Mean sample coverage, also sample completeness, for each taxon based on iNEXT3D (Chao & Hu, 2025).

| **Order** | **Mean sample coverage** |
| --- | --- |
| Coleoptera | 0.90 |
| Heteroptera | 0.67 |
| Lepidoptera | 0.91 |

**Table S 2:** Summary statistics (minimum, maximum, mean and standard deviation) of environmental variables and spatial distances among plots within each study region. Variables include inter-plot distance (km), seasonal mean temperature (°C), direct solar radiation (W m⁻²), and canopy openness.

| Region | Min distance in km | Max distance in km | Mean distance in km ± SD | Min Season. Temp | Max Season. Temp | Mean Season. Temp ± SD | Min solar radiation | Max solar radiation | Mean solar radiation ± SD | Min canopy openness | Max Canopy openness | Mean canopy openness ± SD |
| --- | --- | --- | --- | --- | --- | --- | --- | --- | --- | --- | --- | --- |
| Schorfheide-Chorin | 0.15 | 38.66 | 14.07 ± 7.0. | 15.748 | 16.186 | 15.968 ± 0.110 | 370.133 | 396.087 | 387.624 ± 5.037 | 0.029 | 0.499 | 0.166 ± 0.104 |
| Hainich-Dün | 0.25 | 39.10 | 16.04 ± 9.73 | 14.130 | 15.151 | 14.583 ± 0.251 | 322.676 | 364.313 | 346.518 ± 11.365 | 0.017 | 0.424 | 0.099 ± 0.084 |
| Schwäbische Alb | 0.32 | 20.62 | 9.37 ± 5.05 | 13.592 | 14.412 | 13.880 ± 0.172 | 355.144 | 383.050 | 365.009 ± 7.351 | 0.016 | 0.322 | 0.101 ± 0.066 |
| Steigerwald | 0.06 | 15.96 | 6.63 ± 4.10 | 15.252 | 15.530 | 15.427 ± 0.087 | 362.823 | 374.785 | 370.334 ± 3.324 | 0.018 | 0.271 | 0.099 ± 0.047 |
| Bavarian Forest National Park | 0.08 | 24.69 | 10.59 ± 7.60 | 10.835 | 13.564 | 12.187 ± 0.923 | 334.867 | 349.573 | 340.660 ± 3.754 | 0.022 | 0.926 | 0.332 ± 0.266 |

**Figure S 11:** Responses of raw trait values (mean body size in blue, colour lightness in orange) to environmental parameters.

**Table S 3:** Effects of macro-, and microclimate on morphological trait values logarithmised body size and colour lightness of predominantly diurnal Coleoptera and Heteroptera and nocturnal Lepidoptera. The macroclimatic variable temperature is displayed by seasonal mean temperature, direct radiation is residualised. SE = standard error, AIC: Akaike information criterion.

|  | **Mean body size** | | **Colour lightness** | |
| --- | --- | --- | --- | --- |
| *Predictors* | *Estimates* ± *SE* | *p* | *Estimates* ± *SE* | *p* |
| **Order** | | | | |
| Coleoptera | 0.73 ± 0.01 | **<0.001** | 118.61 ± 1.01 | **<0.001** |
| Heteroptera | 0.16 ± 0.03 | 0.660 | -4.55 ± 1.89 | **0.016** |
| Lepidoptera | 0.41 ± 0.01 | **<0.001** | 23.49 ± 0.74 | **<0.001** |
| **Macroclimate** | | | | |
| Coleoptera: Seasonal mean temperature | -0.10 ± 0.01 | **<0.001** | 0.86 ± 0.89 | 0.332 |
| Heteroptera: Seasonal mean temperature | 0.04 ± 0.03 | 0.123 | 4.60 ± 1.63 | **0.005** |
| Lepidoptera: Seasonal mean temperature | -0.05 ± 0.01 | **<0.001** | 4.94 ± 1.06 | **<0.001** |
| Coleoptera: Residual solar radiation | 0.06 ± 0.01 | **<0.001** | 0.26 ± 0.84 | 0.752 |
| Heteroptera: Residual solar radiation | -0.21 ± 0.05 | **<0.001** | 0.31 ± 2.64 | 0.906 |
| Lepidoptera: Residual solar radiation | 0.01 ± 0.01 | 0.635 | -4.77 ± 0.94 | **<0.001** |
| **Microclimate** | | | | |
| Coleoptera: Canopy openness | -0.04 ± 0.01 | **<0.001** | -1.34 ± 0.57 | **0.020** |
| Heteroptera: Canopy openness | 0.02 ± 0.04 | 0.653 | 0.57 ± 2.02 | 0.777 |
| Lepidoptera: Canopy openness | 0.02 ± 0.02 | 0.270 | -4.84 ± 1.09 | **<0.001** |
| N | 5 _Region_ | | 5 _Region_ | |
|  | 394 _Plot_ | | 394 _Plot_ | |
| Observations | 961 | | 961 | |
| Marginal R^2^ / Conditional R^2^ | 0.285/ NA | | 0.341 / 0.345 | |
| AIC | -2559.174 | | 4985.343 | |

**Table S 4:** Phylogenetic component: Effects of macro-, and microclimate on predicted trait values body size and colour lightness of predominantly diurnal Coleoptera and Heteroptera and nocturnal Lepidoptera. SE = standard error, AIC: Akaike information criterion.

|  | **Predicted mean body size** | | **Predicted colour lightness** | |
| --- | --- | --- | --- | --- |
| *Predictors* | *Estimates* ± *SE* | *p* | *Estimates* ± *SE* | *p* |
| **Order** | | | | |
| Coleoptera | 0.66 ± 0.01 | **<0.001** | 118.87 ± 0.83 | **<0.001** |
| Heteroptera | 0.14 ± 0.03 | **<0.001** | -5.58 ± 1.33 | **<0.001** |
| Lepidoptera | 0.45 ± 0.01 | **<0.001** | 22.35 ± 0.52 | **<0.001** |
| **Macroclimate** | | | | |
| Coleoptera: Seasonal mean temperature | -0.05 ± 0.01 | **<0.001** | -0.78 ± 0.70 | 0.268 |
| Heteroptera: Seasonal mean temperature | 0.06 ± 0.02 | **0.012** | 2.52 ± 1.18 | **0.033** |
| Lepidoptera: Seasonal mean temperature | -0.02 ± 0.01 | 0.107 | 5.63 ± 0.81 | **<0.001** |
| Coleoptera: Residual solar radiation | 0.04 ± 0.01 | **<0.001** | 0.44 ± 0.65 | 0.502 |
| Heteroptera: Residual solar radiation | -0.18 ± 0.04 | **<0.001** | 1.21 ± 1.87 | 0.518 |
| Lepidoptera: Residual solar radiation | 0.01 ± 0.01 | 0.411 | -2.52 ± 0.71 | **<0.001** |
| **Microclimate** | | | | |
| Coleoptera: Canopy openness | 0.01 ± 0.01 | 0.577 | -0.18 ± 0.42 | 0.670 |
| Heteroptera: Canopy openness | 0.06 ± 0.03 | 0.075 | 2.09 ± 1.42 | 0.140 |
| Lepidoptera: Canopy openness | 0.01 ± 0.02 | 0.512 | -4.15 ± 0.78 | **<0.001** |
| N | 5 _Region_ | | 5 _Region_ | |
|  | 394 _Plot_ | | 394 _Plot_ | |
| Observations | 961 | | 961 | |
| Marginal R^2^ / Conditional R^2^ | 0.394 / 0.397 | | 0.553 / 0.559 | |
| AIC | -2871.846 | | 4357.159 | |

**Table S 5:** Specific component: Effects of macro-, and microclimate on residual trait values body size and colour lightness of predominantly diurnal Coleoptera and Heteroptera and nocturnal Lepidoptera. SE = standard error, AIC: Akaike information criterion.

|  | **Residual mean body size** | | **Residual colour lightness** | |
| --- | --- | --- | --- | --- |
| *Predictors* | *Estimates* ± *SE* | *p* | *Estimates* ± *SE* | *p* |
| **Order** | | | | |
| Coleoptera | 0.07 ± 0.00 | **<0.001** | 0.04 ± 0.27 | 0.891 |
| Heteroptera | 0.02 ± 0.01 | 0.066 | 0.70 ± 1.11 | 0.527 |
| Lepidoptera | -0.04 ± 0.01 | **<0.001** | 1.61 ± 0.42 | **<0.001** |
| **Macroclimate** | | | | |
| Coleoptera: Seasonal mean temperature | -0.05 ± 0.00 | **<0.001** | 1.33 ± 0.25 | **<0.001** |
| Heteroptera: Seasonal mean temperature | -0.02 ± 0.01 | **0.049** | 2.17 ± 0.85 | **0.010** |
| Lepidoptera: Seasonal mean temperature | -0.03 ± 0.01 | **<0.001** | -1.64 ± 0.40 | **<0.001** |
| Coleoptera: Residual solar radiation | 0.01 ± 0.01 | **0.019** | -0.28 ± 0.34 | 0.422 |
| Heteroptera: Residual solar radiation | -0.05 ± 0.02 | **0.014** | -0.95 ± 1.50 | 0.525 |
| Lepidoptera: Residual solar radiation | -0.01 ± 0.01 | 0.207 | -2.16 ± 0.42 | **<0.001** |
| **Microclimate** | | | | |
| Coleoptera: Canopy openness | -0.05 ± 0.00 | **<0.001** | -1.38 ± 0.32 | **<0.001** |
| Heteroptera: Canopy openness | -0.04 ± 0.01 | **0.004** | -1.53 ± 1.17 | 0.194 |
| Lepidoptera: Canopy openness | 0.01 ± 0.01 | 0.187 | -1.27 ± 0.61 | **0.037** |
| N | 5 _Region_ | | 5 _Region_ | |
|  | 394 _Plot_ | | 394 _Plot_ | |
| Observations | 961 | | 961 | |
| Marginal R^2^ / Conditional R^2^ | 0.040 / 0.045 | | 0.018 / 0.022 | |
| AIC | -4373.151 | | 3969.398 | |

**Table legends**

[**Table S 1:** Mean sample coverage, also sample completeness, for each taxon based on iNEXT3D (Chao & Hu, 2025).	11](#_Toc226906217)

[**Table S 2:** Summary statistics (minimum, maximum, mean and standard deviation) of environmental variables and spatial distances among plots within each study region. Variables include inter-plot distance (km), seasonal mean temperature (°C), direct solar radiation (W m⁻²), and canopy openness.	12](#_Toc226906218)

[**Table S 3:** Effects of macro-, and microclimate on morphological trait values logarithmised body size and colour lightness of predominantly diurnal Coleoptera and Heteroptera and nocturnal Lepidoptera. The macroclimatic variable temperature is displayed by seasonal mean temperature, direct radiation is residualised. SE = standard error, AIC: Akaike information criterion.	13](#_Toc226906219)

[**Table S 4:** Phylogenetic component: Effects of macro-, and microclimate on predicted trait values body size and colour lightness of predominantly diurnal Coleoptera and Heteroptera and nocturnal Lepidoptera. SE = standard error, AIC: Akaike information criterion.	14](#_Toc226906220)

[**Table S 5:** Specific component: Effects of macro-, and microclimate on residual trait values body size and colour lightness of predominantly diurnal Coleoptera and Heteroptera and nocturnal Lepidoptera. SE = standard error, AIC: Akaike information criterion.	15](#_Toc226906221)

**Figure legends**

[**Figure S 1:** Schematic overview of the responses of body size and colour lightness to temperature according to the *Heat Conservation* and *Thermal Melanism Hypotheses*.	1](#_Toc226906834)

[**Figure S 2:** Schematic overview of the responses of body size and colour lightness to solar radiation according to the *Heat Transfer Theory* and *Bogert’s Rule*.	2](#_Toc226906835)

[**Figure S 3:** Relationship between measured body size of individual specimens and mean body size for Lepidoptera. There is a strong correlation between individual body size and species mean (R^2^ = 0.96, p-value < 0.001). The blue line represents the linear regression, red triangles highlight *B. betularia*.	5](#_Toc226906836)

[**Figure S 4:** Relationship between measured colour lightness of individual specimens and mean colour lightness for Lepidoptera. There is a strong correlation between individual colour lightness and species mean (R^2^ = 0.90, p-value < 0.001). The blue line represents the linear regression, red triangles highlight *B. betularia*.	5](#_Toc226906837)

[**Figure S 5:** Individuals of *Biston betularia* (18-22) depicted in “The Macrolepidoptera of Germany” (Segerer et al., 2011).	6](#_Toc226906838)

[**Figure S 6:** Relationship between measured body size of individual specimens and mean body size for saproxylic Coleoptera. There is a strong correlation between individual trait and species mean (R^2^ = 0.96, p-value < 0.001). The blue line represents a linear regression. This dataset was not used in our study but is displayed for demonstration purposes.	6](#_Toc226906839)

[**Figure S 7:** Morphological trait correlation between mean body size and colour lightness for the 1555 species of predominantly diurnal Coleoptera (left), the 183 species of predominantly diurnal Heteroptera (middle) and the 454 species of nocturnal Lepidoptera (right) detected in the five study regions. The scale of the y-axes is logarithmic.	7](#_Toc226906840)

[**Figure S 8:** Schematic overview of the calculation of Direct Radiation. MOD06 and MYD06 refer to the corresponding products of the MODIS sensor. The R package ‘terra’ was used to calculate the hourly direct radiation and sun angle values.	9](#_Toc226906841)

[**Figure S 9:** Correlation plot displaying the relationship between the climatic parameters and the residualised variable solar radiation.	10](#_Toc226906842)

[**Figure S 10:** Density histogram of scaled seasonal mean temperature (left), scaled direct solar radiation (middle), and scaled canopy openness (right) for the five study regions.	10](#_Toc226906843)

[**Figure S 11:** Responses of raw trait values (mean body size in blue, colour lightness in orange) to environmental parameters.	13](#_Toc226906844)

**References**

Bates, D., Maechler, M., Bolker, B., & Walker, S. (2021). lme4: Linear Mixed-Effects Models using ‘Eigen’ and S4. <https://cran.r-project.org/web/packages/lme4/lme4.pdf>. Retrieved from <https://cran.r-project.org/web/packages/lme4/lme4.pdf>

Battisti, A., Marini, L., Pitacco, A., & Larsson, S. (2013). Solar radiation directly affects larval performance of a forest insect. *Ecological Entomology*, *38*(6), 553–559. <https://doi.org/10.1111/een.12047>

Bishop, T. R., Robertson, M. P., Gibb, H., van Rensburg, B. J., Braschler, B., Chown, S. L., . . . Pincheira‐Donoso, D. (2016). Ant assemblages have darker and larger members in cold environments. *Global Ecology and Biogeography*, *25*(12), 1489–1499. <https://doi.org/10.1111/geb.12516>

Chao, A., & Hu, K.‐H. (2025). Interpolation and Extrapolation for Three Dimensions of Biodiversity [R package iNEXT.3D version 1.0.8] (Version 1.0.8) [Computer software]. Comprehensive R Archive Network (CRAN): Comprehensive R Archive Network (CRAN). Retrieved from <https://cran.r-project.org/web/packages/iNEXT.3D/index.html>

Clusella-Trullas, S., van Wyk, J. H., & Spotila, J. R. (2007). Thermal melanism in ectotherms. *Journal of Thermal Biology*, *32*(5), 235–245. <https://doi.org/10.1016/j.jtherbio.2007.01.013>

GDAL/OGR contributors (2024). GDAL/OGR Geospatial Data Abstraction software Library. [Computer software].

Heidrich, L., & Müller, J. (2023a). Phylogeny of moths. Version 4. Biodiversity Exploratories Information System. Dataset. <https://www.bexis.uni-jena.de/ddm/data/Showdata/31608?version=4>. Retrieved from <https://www.bexis.uni-jena.de/ddm/data/Showdata/31608?version=4>

Heidrich, L., & Müller, J. (2023b). Phylogeny of true bugs. Version 3. Biodiversity Exploratories Information System. Dataset. <https://www.bexis.uni-jena.de/ddm/data/Showdata/31607?version=3>. Retrieved from <https://www.bexis.uni-jena.de/ddm/data/Showdata/31607?version=3>

Heidrich, L., Pinkert, S., Brandl, R., Bässler, C., Hacker, H., Roth, N., . . . Friess, N. (2021). Noctuid and geometrid moth assemblages show divergent elevational gradients in body size and color lightness. *Ecography.* Advance online publication. <https://doi.org/10.1111/ecog.05558>

Hijmans, R. J., Barbosa, M., Bivand, R., Brown, A., Chirico, M., Cordano, E., . . . Sumner, M. D. (2025). *Spatial Data Analysis [R package terra version 1.8-29]*. Comprehensive R Archive Network (CRAN). Retrieved from <https://cran.r-project.org/web/packages/terra/index.html>

Müller, J., Heibl, C., & Heidrich, L. (2019). Phylogeny of Coleoptera species used in the TreeScape Project, 2007-2016. Version 2. Biodiversity Exploratories Information System. Dataset. <https://www.bexis.uni-jena.de/ddm/data/Showdata/25686?version=2>. Retrieved from <https://www.bexis.uni-jena.de/ddm/data/Showdata/25686?version=2>

Platnick, S., Meyer, K. G., King, M. D., Wind, G., Amarasinghe, N., Marchant, B., . . . Riedi, J. (2017). The MODIS cloud optical and microphysical products: Collection 6 updates and examples from Terra and Aqua. *IEEE Transactions on Geoscience and Remote Sensing*, *55*(1), 502–525. <https://doi.org/10.1109/TGRS.2016.2610522>

Porter, W. P., & Gates, D. M. (1969). Thermodynamic Equilibria of Animals with Environment. *Ecological Monographs*, *39*(3), 227–244. <https://doi.org/10.2307/1948545>

Segerer, A., Hausmann, A., Behounek, G., Speidel, W., & Witt, T. J. (2011). *The Macrolepidoptera of Germany* (1. Edition). Budapest: Goecke & Evers.
